# Flavonoids-Rich *Orthosiphon stamineus* Extract as New Candidate for Angiotensin I-Converting Enzyme Inhibition: A Molecular Docking Study

**DOI:** 10.3390/molecules21111500

**Published:** 2016-11-09

**Authors:** Armaghan Shafaei, Md Shamsuddin Sultan Khan, Abdalrahim F. A. Aisha, Amin Malik Shah Abdul Majid, Mohammad Razak Hamdan, Mohd Nizam Mordi, Zhari Ismail

**Affiliations:** 1 Department of Pharmaceutical Chemistry, School of Pharmaceutical Sciences, Universiti Sains Malaysia, Minden, Penang 11800, Malaysia; armaghn.shafaei@gmail.com (A.S.); abedaisheh@yahoo.com (A.F.A.A.); 2 EMAN Testing & Research Laboratory, Department of Pharmacology, School of Pharmaceutical Sciences, Universiti Sains Malaysia, Minden, Penang 11800, Malaysia; jupitex@gmail.com (M.S.S.K.); aminmalikshah@usm.my (A.M.S.A.M.); 3 Centre for Drug Research, Universiti Sains Malaysia, Minden, Penang 11800, Malaysia; ikraqlah@gmail.com (M.R.H.); mnizam@usm.my (M.N.M.)

**Keywords:** angiotensin-converting enzyme, hypertension, flavonoids, HPLC-UV, molecular ducking study

## Abstract

This study aims to evaluate the in vitro angiotensin-converting enzyme (ACE) inhibition activity of different extracts of *Orthosiphon stamineus* (OS) leaves and their main flavonoids, namely rosmarinic acid (RA), sinensetin (SIN), eupatorin (EUP) and 3′-hydroxy-5,6,7,4′-tetramethoxyflavone (TMF). Furthermore, to identify possible mechanisms of action based on structure–activity relationships and molecular docking. The in vitro ACE inhibition activity relied on determining hippuric acid (HA) formation from ACE-specific substrate (hippuryl-histidyl-leucine (HHL)) by the action of ACE enzyme. A High Performance Liquid Chromatography method combined with UV detection was developed and validated for measurement the concentration of produced HA. The chelation ability of OS extract and its reference compounds was evaluated by tetramethylmurexide reagent. Furthermore, molecular docking study was performed by *LeadIT-FlexX*: BioSolveIT’s LeadIT program. OS ethanolic extract (OS-E) exhibited highest inhibition and lowest IC_50_ value (45.77 ± 1.17 µg/mL) against ACE compared to the other extracts. Among the tested reference compounds, EUP with IC_50_ 15.35 ± 4.49 µg/mL had highest inhibition against ACE and binding ability with Zn (II) (56.03% ± 1.26%) compared to RA, TMF and SIN. Molecular docking studies also confirmed that flavonoids inhibit ACE via interaction with the zinc ion and this interaction is stabilized by other interactions with amino acids in the active site. In this study, we have demonstrated that changes in flavonoids active core affect their capacity to inhibit ACE. Moreover, we showed that ACE inhibition activity of flavonoids compounds is directly related to their ability to bind with zinc ion in the active site of ACE enzyme. It was also revealed that OS extract contained high amount of flavonoids other than RA, TMF, SIN and EUP. As such, application of OS extract is useful as inhibitors of ACE.

## 1. Introduction

Hypertension or high blood pressure is one of the main causes of death in industrialized societies [1]. Globally, about one-quarter of the adult population suffers from hypertension [2]. It is a major risk factor for cardiovascular disease and related complications, such as heart disease, kidney damage, eye damage and stroke [3]. Lifestyle modifications, including changes in dietary habits, might avoid, delay or reduce the need for medication [4].

The renin-angiotensin–aldosterone system (RAAS) is a key factor in the maintenance of arterial blood pressure. Angiotensin-converting enzyme (ACE) (EC3.4.15.1) is one of the main components of the RAAS [5]. ACE is a glycosylated zinc dipeptidyl-carboxypeptidase, and its main function is catalyzing the conversion of the precursor angiotensin I into angiotensin II. ACE is a peptide responsible for triggering vasoconstrictive effects to regulate arterial blood pressure and electrolyte balance and degrades bradykinin, which is a potent vasodilator in RAAS. Therefore, inhibition of ACE has become a promising way for regulation and treatment of high blood pressure [6].

Although nowadays many synthetic ACE inhibitors such as captopril, benazepril, enlapril and alacepril are widely used in the treatment of hypertension and heart failure, the chronic use of these synthetic inhibitors may be associated with many undesirable side effects, such as persistent cough, postural hypotension, renal failure, and angioedema [7,8,9]. Recently, many studies have been carried out to discover new ACE inhibitor compounds from natural resources [10,11]. Natural ACE inhibitors as alternatives to synthetic ones have great interest among researchers for their better drug profiles and fewer side effects [12]. The ACE inhibition of natural substances, such as peptides and triterpenes, has been described in the literature [13,14,15]. Recent studies also demonstrated beneficial in vitro ACE inhibition effect of flavonoids compounds isolated from different plants [16,17,18]. These beneficial effects have largely been ascribed to the generation of chelate complexes within the active center of ACE [19]. Phenolic compounds such as ferulic acid and tannic acid and flavonoids such as quercetin, anthocyanins, flavones and flavonols have shown to exhibit a capacity to inhibit different zinc metalloproteinases, including ACE [19,20,21,22,23,24,25]. Moreover, the ACE-inhibitory (ACE-I) activity of different foods and plant extracts rich in flavonoids have also been demonstrated by in vitro and in vivo studies in spontaneous hypertensive rats [16,26,27,28]. However, only a few studies have been done to address the relationship between the ACE-I activity of flavonoids compounds and their structures [12,14].

There are several methods that have been proposed for the measurement of ACE activity such as spectrophotometry, High-performance liquid chromatography (HPLC), fluorimetry and micellar electrokinetic chromatography [29,30,31]. Among the methods, spectrophotometry and HPLC methods are employed most frequently [29]. The spectrophotometric method developed by Cushman and Cheung (1971) is the most commonly utilized in vitro assay [32]. This method uses the ACE-specific substrate hippuryl-histidyl-leucine (HHL) coupled with spectrophotometric detection of the product hippuric acid (HA). This method relies on the principle of determining hippuric acid formation from HHL by the action of ACE, resulting in the removal of C-terminal dipeptide histidylleucine. However, ethyl acetate extraction procedure in spectrophotometry method can also extract unhydrolyzed HHL apart from HA, therefore incorrect positive results cannot be excluded [33]. Alternatively, HPLC, as an analytical technique, has provided specific quantification of compounds at low concentrations. Moreover, the accuracy and reproducibility than spectrophotometry makes it well suited for quantitative study of enzymatic-catalyzed reaction that those not require a chromophore or radiolabeling [34]. 

*Orthosiphon stamineus* Benth. (OS) or misai kucing is a plant from Lamiaceae family. It is a medicinal plant and native in Southeast Asia. The leaves of this plant have been reported to have anti-diabetes, anti-hypertension, diuretic and anti-cancer effects [35,36,37,38]. OS leaves have been reported to contain high content of phenolic acid such as rosmarinic acid (RA) and flavonoids contents, such as sinensetin (SIN), eupatorin (EUP) and 3′-hydroxy-5,6,7,4′-tetramethoxyflavone (TMF) [39]. Therefore, this plant is an important candidate to develop a drug with ACE inhibition activity. Therefore, the objectives of this study are to evaluate the in vitro ACE inhibition activity of different extracts of OS leaves, three flavonoids sinensetin (SIN), eupatorin (EUP) and 3′-hydroxy-5,6,7,4′-tetramethoxyflavone (TMF) belonging to lipophilic flavones and a caffeic acid derivative, phenolic rosmarinic acid (RA), by developing a new HPLC-UV method. Moreover, this study investigates the chelation ability of Zinc (II) Ion by the main flavonoids and phenolic compounds presence in OS extracts. The results will be used to identify possible mechanisms of action based on structure–activity relationships and molecular docking.

## 2. Results

### 2.1. Identification and Quantification of RA, TMF, SIN and EUP in OS Extracts

**Figure 1:**
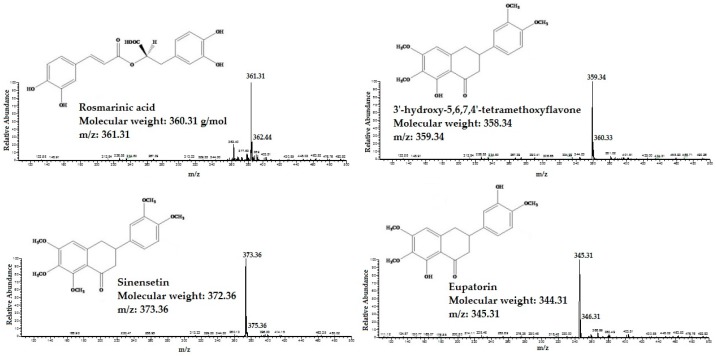
The High performance liquid chromatography-mass spectrometry (HPLC/MS) spectra of standard compounds: 3′-hydroxy-5,6,7,4′-tetramethoxyflavone, eupatorin, sinensetin and rosmarinic acid.

The HPLC/MS was applied for identifcation of RA, TMF, SIN and EUP in different extracts of OS. The standard compounds of RA, TMF, SIN and EUP were analyzed in both negative and positive ESI mode. The results showed that ESI in positive mode was sensitive to all tested compounds. All four standard compounds were well detected. The detected constituents all exhibited their quasi-molecular ions [M − H]^+^ (Figure 1).

**Figure 2:**
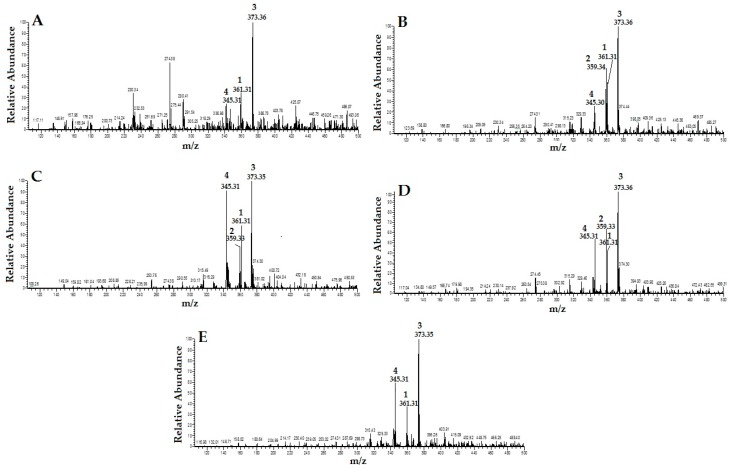
HPLC/MS spectra of different extracts of *Orthosiphon stamineus* (OS) (1: RA; 2: TMF; 3: SIN; and 4: EUP): (**A**) water extract (OS-W); (**B**) ethanolic extract of (OS-E); (**C**) methanolic extract of (OS-M); (**D**) 50% ethanolic extract (OS-EW); and (**E**) 50% methanolic extract (OS-MW).

By carefully studying the mass spectra of standard compounds and comparing them with the mass spectra of different extracts of OS, the peak related to the standard compounds in the extracts were designated and identified (Figure 2). They were: (1) RA; (2) TMF; (3) SIN; and (4) EUP. As shown in Figure 2, TMF (2) was not detected in OS-W and OS-MW samples.

**Figure 3:**
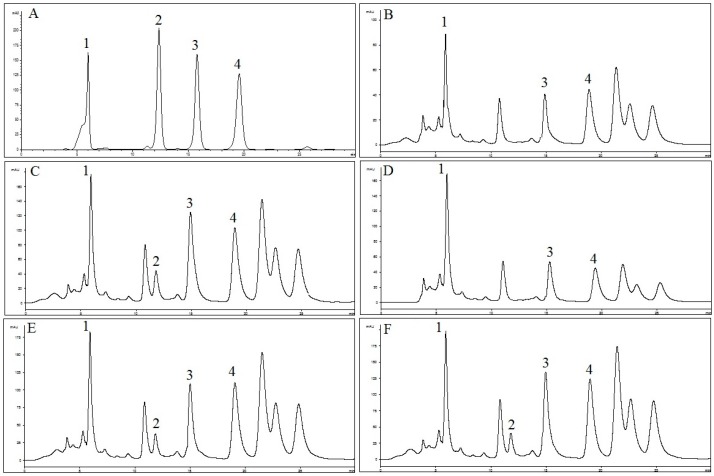
HPLC chromatograms of: (**A**) reference compounds (1: RA; 2: TMF; 3: SIN; and 4: EUP); (**B**) OS-W; (**C**) OS-E; (**D**) OS-M; (**E**) OS-EW; and (**F**) OS-MW at 330 nm.

The HPLC method was applied for quantification of RA, TMF, SIN and EUP in OS extracts. As shown in Figure 3, selected standard compounds were well separated by the described HPLC method. In Table 1, concentrations of RA, TMF, SIN and EUP determined by HPLC in different extracts of OS are presented. Results were derived from the mean of peak area from three replicates injections. The results show that concentration of RA in OS-EW (235.5 ± 0.2 mg/g) and concentrations of TMF, SIN and EUP (11.5 ± 1.0, 23.2 ± 0.1 and 166.9 ± 3.5 mg/g) in OS-E were higher compared to other extracts. Results from this study are in line with previous reports suggesting that lipophilic flavones (TMF, SIN and EUP) are the major bioactive constituents in OS-E [40].

### 2.2. Validation of HPLC Method for Measurement of ACE Inhibition Activity

In this work, the analyses were carried out by HPLC-UV, and HHL was used as substrate. The choice of the substrate was based on the sensitivity (corresponding detection limits have been estimated), the cost and more importantly the ACE amounts required for hydrolysis [29]. Under the aforementioned HPLC conditions, complete base line separation of HA and HHL was achieved on a Zorbax Eclipse plus phenyl-hexyl column. HA and HHL were eluted at 4.9 min and 8.3 min, respectively. The total time for the separation of HHL and HA was 10 min. For ACE reaction mixture, elution profile of HA and HHL was similar to the observed ones for their corresponding standards. Linearity of the method was evaluated by determining a series of seven working standards solution of HA in three replicates. 

Different concentrations of ACE enzyme (0.05–0.00157 U/mL) were reacted with HHL to measure produced HA. A linear regression equation and correlation coefficient were established from the graph by plotting the mean of three replicates peak areas of HA versus the HA concentration and three replicates peak areas of HA versus ACE concentration. It was found that the HA solution was linear over the evaluated concentration range with R^2^ > 0.999. The sensitivity of the method was evaluated by Limit of Detection (LOD) and Limit of Quantification (LOQ) analyses. The values of LOD and LOQ for HA were 0.14 ± 0.00 and 0.44 ± 0.00 µg/mL, respectively. The peak areas were used to calculate relative standard deviation (%RSD) of the reference compounds, and intra-day and inter-day precision rates. The variations were found to be in the range of 97.71%–102.28% for HA with RSD less than 2%. For recovery rates, they ranged between 99.00% and 101.7%, with RSD less than 3%, which indicates that the applied method is reproducible.

### 2.3. In Vitro ACE Inhibition Activity

**Figure 4:**
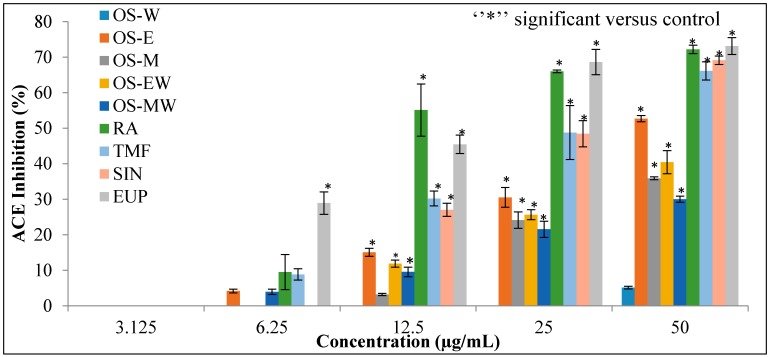
The dose–response relationship of OS extracts and reference compounds on in vitro ACE inhibitory assay. Data are presented as mean ± SD, (*p* < 0.05).

Different extracts of OS and compounds of RA, TMF, SIN and EUP were evaluated for the substantive experiment of the rapid in vitro assay of ACE inhibition. The results of ACE inhibitory activity of OS extracts and reference compounds at the concentration range of (3.125–50 µg/mL) are shown in Figure 4. Captopril, a synthetic ACE-I, was used as a positive control. The half maximal inhibitory concentration (IC_50_) value is deduced from the linear regression equation obtained by plotting percentage ACE inhibition activity versus concentration. Table 2 depicts IC_50_ values of the extracts and positive control. The OS-E exhibits highest inhibition and lowest IC_50_ value (45.77 ± 1.17 µg/mL) against ACE compared to the other extracts. The ACE inhibitory activity of all extracts follows the order: OS-E > OS-M > OS-EW > OS-MW > OS-W. Among the reference compounds, EUP showed the highest inhibition lowest IC_50_ (15.35 ± 4.49 µg/mL) value.

### 2.4. Chelation of Zinc (II) Ion by OS-E, RA, TMF, SIN, EUP and Captopril

**Figure 5:**
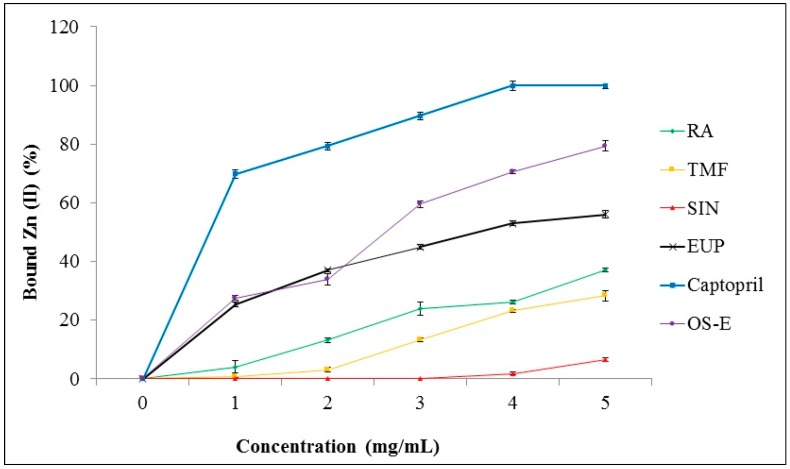
Zn (II) chelating activity of OS-E, RA, TMF, SIN, EUP and captopril. Data are presented as mean ± SD.

Among the tested extracts, OS-E showed highest ACE inhibition activity. Therefore, this extract was selected to study its chelation ability with zinc (II) ion. Zn (II) chelating activity of OS-E, RA, TMF, SIN, EUP and captopril was determined by tetramethylmurexide (TMM) reagent. In the first step, a standard curve was constructed from the absorption of TMM and complexes of metal ions versus the concentration range of metal ions (0–3.2 mmol/mL) at 462 nm. Based on this standard curve, the amount of metal ions bound by the compound tested was evaluated. The results demonstrated that captopril was the best chelator with Zn (II) (100% ± 1.59% at concentration of 4 mg/mL) (Figure 5). OS-E extract also showed a high ability (79.42 ± 1.91) to bind with Zn (II) at final concentration of 5 mg/mL. Among the standard compounds, EUP demonstrated the highest bounding ability with Zn (II) (56.03% ± 1.26% at concentration of 5 mg/mL) compared to RA, TMF and SIN. SIN showed the lowest bounding ability with Zn (II) (6.71% ± 0.62% at concentration of 5 mg/mL) in comparison to the other tested compounds.

### 2.5. Molecular Docking Study

The docking scores and binding affinities of RA, SIN, TMF, EUP and captopril compounds with the active site of ACE, which is Zn (II), were evaluated (Table 3). Captopril, as a well-known ACE inhibitor, showed the highest binding affinity energy (ΔG) and ligand efficiency with −38 kJ/mol and 0.64 kcal/mol per heavy atom, respectively. Among tested compounds, EUP had the highest binding affinity energy (ΔG) and ligand efficiency with −29 kJ/mol and 0.28 kcal/mol per heavy atom, respectively. After EUP, RA showed to have high possibility to bind with an active site of ACE with −17 kJ/mol binding affinity energy and 0.16 kcal/mol per heavy atom ligand efficiency, whereas TMF, with −6 kJ/mol and 0.06 kcal/mol per heavy atom, respectively, showed low binding affinity energy and ligand efficiency. In addition, SIN did not show any interaction with zinc ion in the active center of ACE.

**Figure 6:**
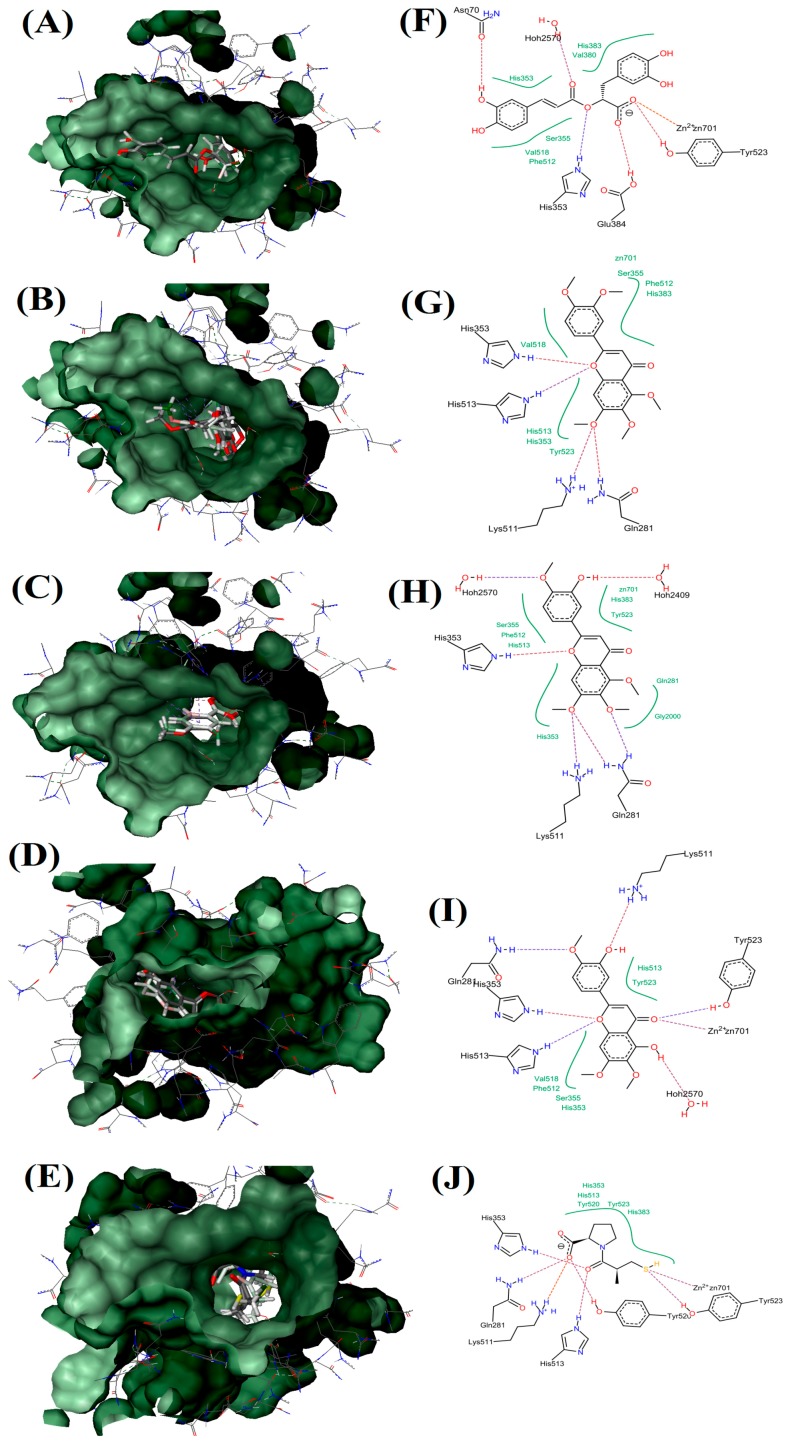
Visualization of ACE and OS extracts reference compounds interaction profile: Surface visualization of proteins in ACE with the ligands: RA (**A**); SIN (**B**); TMF (**C**); EUP (**D**); and Captopril (**E**). Active site residues interaction of protein in ACE with the ligands: RA (**F**); SIN (**G**); TMF (**H**); EUP (**I**); and Captopril (**J**). Hydrophobic interaction shown in green region.

Docking studies of flavonoids indicated that these compounds are able to inhibit ACE via interaction with zinc ion and this interaction is stabilized by other interactions with amino acids in the active site [6]. As shown in Figure 6, captopril is able to interact with zinc ion directly by its sulfhydryl group which forms a coordinate covalent bond with the metal ion. Moreover, strong hydrogen bonds between His513, His353 and the central carbonyl group adhered to ACE molecule on one site, while one of the carboxyl groups on the terminal proline interacts with the residues Tyr 520, Gln 281, and Lys 511 at the other site of ACE molecule. Furthermore, EUP and RA are able to interact with zinc ion in the active site of ACE via their ketone group (in the C4 at the C-ring) and carboxyl group, respectively. Moreover, EUP interacted with amino acid residues such as Tyr523, Hoh2570, His513, His353, Gln281 and Lys511 with its 3 hydroxy in B-ring, 5 hydroxy in A-ring, oxygen atom and ketone group at the C-ring via seven hydrogen bonds. While RA showed six hydrogen bonds via its acrylic acid and carboxyl groups with amino acid residues (Tyr523, Glu384, His353, Asn70 and Hoh2570). Concerning TMF and SIN, neither of these compounds interacted with zinc in the active site. However, these compounds formed interactions with amino acids in the active site of ACE but their binding affinity energy and ligand efficiency were very low (Table 3).

## 3. Discussion

Flavonoids compounds obtained from the plant extracts as ACE inhibitor has gained more interest during the last 10 years [6]. Many in vitro studies demonstrated the effect flavonoids as ACE inhibitors [16,26]. The beneficial effects of flavonoids have largely been explained by generation of chelate complexes within the active center of ACE [19,26]. Basically, flavonoids are polyphenolic molecules at low molecular weight with the basic structure of two phenyl rings (A and C rings) joined with three carbons that make a closed pyran ring structure (B ring) [41,42] (Figure 1). Furthermore, based on their structural differences, flavonoids can be divided into several subfamilies such as flavanones, flavones, flavonols, isoflavones, flavanols (essentially, flavan-3-ols) and anthocyanidins based on their degree of unsaturation and oxidation of the oxygenated heterocycle [41,43].

Previous studies have shown that flavonoid structure has played an important role in its biological function such as cytotoxicity, antioxidant and antiproliferative activities [44,45]. Moreover, several studies have shown that flavonoids are able to exhibit zinc metalloproteinases such as ACE and this activity depends on the flavonoids structure [46,47]. Although in vitro and in vivo ACE inhibition activity of some flavonoids such as quercetin and phenolics such as ferulic acid and tannic acid have been studied [20,21], the data on ACE inhibition and therefor blood pressure-reducing potency of many more flavonoids, especially those that are present in OS (a plant that has been used traditionally for treating hypertension), have not yet been studied. Therefore, in this study ACE inhibitory properties of different extracts of OS and pure phenolic acid (RA) and flavonoids (TMF, SIN and EUP) compounds that are presence in OS extract were evaluated. Moreover, the chelating activity of these compounds with Zn (II) was investigated by TMM reagent. Together with the obtained results, structure–activity relationship analysis and molecular docking were used for a better understanding of how these flavonoids compounds interact with the ACE enzyme.

The results obtained from the inhibitory effects on ACE activity of OS extracts showed that OS-E extract, which contained highest contents of flavonoids (TMF, SIN and EUP) (Table 1), exhibited highest inhibition compared to other extracts. In addition, OS-E extract, which contains a mixture of flavonoids, showed high chelating ability with Zn (II) and it was greater than RA, TMF, SIN or EUP alone (Figure 5). With regard to the flavonoids compounds that have been used in this study, it could be seen that presence of certain functional groups such as hydroxyl, carboxyl, and acrylic acid groups, which can act as hydrogen bond acceptors or donors, have increased the potency to inhibit ACE [6,16]. As shown in Figure 6, EUP produced more number of hydrogen bonds (seven) with the active site of ACE enzyme and therefore showed the highest ACE inhibition compared to other tested compounds, whereas RA contained more number of hydroxyl groups compared to EUP but produced less hydrogen bonds (six) compared to EUP. Restricted docking between RA and the active site of ACE failed, as a consequence of the steric hindrance. In addition, SIN was not able to produce hydrogen bond, due to the lack of suitable functional groups such as hydroxyl, carboxyl, and acrylic. This finding is in line with the in vitro ACE results. EUP showed the lowest IC_50_ value (15.35 ± 4.49 µg/mL) and highest bounding ability with Zn (II) (56.03% ± 1.26% at concentration of 5 mg/mL) compared to RA, TMF and SIN. The in vitro ACE inhibition activity and binding ability with Zn (II) of tested compounds follows the order: EUP > RA > TMF > SIN. Similarly, protein–ligand docking studies also showed the same order: EUP > RA > TMF > SIN.

In this study, we have demonstrated that changes in flavonoids active core affect their capacity to inhibit ACE. Moreover, we showed that ACE inhibition activity of flavonoids compounds is directly related to their ability to bind with zinc ion in the active site of ACE enzyme. In Figure 3, it can be seen that, in addition to the other four discussed compounds (RA, TMF, SIN and EUP), other compounds are present in the extracts and they might also contribute to the ACE inhibition activity. According to Siddiqui and Ismail, OS extract contained high amounts of flavonoids other than RA, TMF, SIN and EUP [48]. Therefore, the unknown compounds can be studied in future research. As such, application of OS extract is useful as inhibitors of ACE.

## 4. Materials and Methods

### 4.1. Chemicals and Reagents

ACE enzyme (from rabbit lung), hippuryl-histidyl-leucine (HHL), hippuric acid (HA), captopril, zinc chloride, tetramethylmurexide and hexamine/HCl were purchased from Sigma-Aldrich (Subang Jaya, Selangor, Malaysia). Rosmarinic acid (RA), Sinensetin (SIN), eupatorin (EUP) and 3′-hydroxy-5,6,7,4′-tetramethoxy flavone (TMF) standards were purchased from Indofine (Hillsborough, NJ, USA). Acetonitrile (HPLC grade), formic acid (HPLC grade) methanol (HPLC grade), orthophosphoric acid (HPLC grade), methanol (AR grade), ethanol (AR grade) and ammonium dihydrogen phosphate, monobasic dihydrogenphosphate and dibasic monohydrogen phosphate were obtained from Merck (Petaling Jaya, Selangor, Malaysia). Deionized water for HPLC was prepared using ultra-pure water purifier system (Elgastat, Bucks, UK).

### 4.2. Plant Material and Extraction

In the current study, the leaves of *Orthosiphon stamineus* (OS) were purchased from Herbagus Sdn. Bhd. Penang-Malaysia and identified by Mr. Shunmugam from the school of Biological Sciences, University Sains Malaysia. A voucher specimen (no. 11009) was deposited at the herbarium of School of Biological Sciences, University Sains Malaysia. The leaves were oven-dried at 40 °C and then pulverized into fine powder using a milling machine (Retsch GmbH, Haan, Germany). In preparing extracts, 100 g powder was extracted with 500 mL of water (OS-W), ethanol (OS-E), methanol (OS-M), 50% ethanol (OS-EW) and 50% methanol (OS-MW) using maceration method for 48 h at 50 °C. After cooling, the extracts were filtered using Whatman filter paper No. 1 (Whatman, Kent, England), concentrated at 50 °C under vacuum using a rotary evaporator (RE121 Buchi, Flawil, Switzerland), and dried using a freeze-drying system (Labconco, Kansas City, MO, USA).

### 4.3. Identification and Quantification of RA, TMF, SIN and EUP in OS Extracts

#### 4.3.1. HPLC Mass Spectrometry

The High Performance Liquid Chromatography Mass Spectrometry (HPLC/MS) was used for identification of RA, TMF, SIN and EUP in different extracts of OS. An Accela Thermo Finnigan LCQ DUO (Mundelein, IL, USA) system equipped with an ESI source was used in this study. The standard compounds of RA, TMF, SIN and EUP were prepared separately in MS grade methanol and 20 μL of each compound was injected through HPLC into ESI probe. The operating conditions were as follows: Colum; Poroshell 120 EC-C18, 2.7 μm (Agilent, Santa Clara, CA, USA), column dimension 2.1 × 100 mm, part number: USCGC 01268; mobile phase, methanol and 0.1% formic acid (90:10) at a flow rate 0.3 mL/min; the capillary temperature, 220 °C; capillary voltage, 4 kV; drying gas (N_2_) flow, 12 L/min; nebulizer (N_2_) pressure, 35 pis. Full scan data acquisition was performed from *m*/*z* 105 to 500 units in MS scan mode.

#### 4.3.2. HPLC

The High Performance Liquid Chromatography (HPLC) analysis was used for quantification of RA, TMF, SIN and EUP in different extracts of OS. HPLC analysis was performed using an Agilent Technologies (Series 1200 infinity, Frankfurt, Germany) system. Separations were accomplished on Nucleosil C18 column (250 × 4.6 mm internal diameter × 5 µm particles size) (Macherey Nagel, Düren, Germany) and column temperature was marinated at 25 °C and the injection sample (20 µL) was eluted at isocratic system comprising of methanol: tetrahydrofuran: water (0.1% H_3_PO_4_) mixture in the volume ratio 55: 5: 40. Flow rate was 0.7 mL/min and detector was set at 330 nm [48].

### 4.4. In Vitro ACE Inhibition Activity

Assay for measuring ACE inhibitory activity was described by Chushman and Cheung (1971) [32]. This method relies on determining hippuric acid (HA) formation from ACE-specific substrate hippuryl-histidyl-leucine (HHL) by the action of ACE enzyme, resulting in the removal of C-terminal dipeptide histidylleucine.

#### 4.4.1. HPLC Method for Measurement of ACE Inhibition Activity

##### Chromatographic Condition

The high performance liquid chromatography (HPLC) was performed using an Agilent Technologies (Series 1200 infinity, Frankfurt, Germany) system. Separations were accomplished on a Zorbax Eclipse plus phenyl-hexyl (4.6 mm × 250 mm, 5 µm, Agilent) and the column temperature was maintained at 30 °C. Substrates of HA and HHL were detected at 228 nm. The flow rate was set at 0.6 mL/min and injection volume was 10 μL. The mobile phase consisted of 20% acetonitrile and 80% of 0.1% ammonium dihydrogen phosphate buffer was used in this analysis with 10 min separation time.

##### HA Calibration Curve

To construct a calibration curve, a series of HA solutions (0.488–31.25 µg/mL) were prepared in water. The calibration was constructed by plotting the relative peak area of the HA versus concentration of HA in triplicate at 7 different concentrations.

##### ACE Calibration Curve

For substrate HHL hydrolysis catalyzed by purified rabbit ACE enzyme, the total reaction volume was 200 µL. Phosphate buffer (pH 8.3) was used to dilute the substrate HHL and rabbit lung ACE enzyme. To 100 µL of 10 mM HHL substrate, 100 µL of ACE enzyme solution with various concentrations was added (0.05–0.00157 U/mL). The reaction mixtures were incubated for 2 h at 37 °C. The reaction was terminated by heating the samples at 100 °C in water bath, and then 800 µL of water was added to all samples, and they were analyzed by stated HPLC method.

##### Validation of HPLC Method

The developed HPLC method was validated in terms of accuracy, precision, linearity range, limit of detection (LOD), and limit of quantification (LOQ) according to the ICH guideline [49]. Accuracy of the HPLC method was evaluated through recovery studies by adding known amounts of HA solution (25, 12.5, 6.25, 3.125 and 1.5625 µg/mL) into the mixture of HA and HHL at 25 µg/mL. The spiked HA solutions were injected three times and the recovery was calculated with the value of detected versus added amounts. The intra-day and inter-day precisions were determined for evaluation of method precision. The intra-day precision was evaluated by injecting seven different concentrations of the standards five times for one day to HPLC system, while inter-day precision was obtained by injecting seven different concentrations of the standards one time for five consecutive days. The resulting peak area was used to calculate standard deviation (SD) and the relative standard deviation (%RSD). The linearity of methods was constructed using calibration curve of HA and ACE over different concentrations in triplicate. LOD and LOQ were measured based on the standard deviation of the response and the slope was assessed according to the following equations:

LOD = 3.3σ/S
(1)

LOQ= 10σ/S
(2)

where σ is the standard deviation of the response and S is the slope of the calibration curve.

### 4.5. In Vitro ACE Inhibition Activity

#### Sample Preparation with the Purified Rabbit Lung ACE

Captopril was used as positive control in this assay at different concentrations (0.0020–0.0625 µM). RA, TMF, SIN and EUP as reference compounds were also used (1.5625–50 µg/mL). HHL as substrate was prepared freshly in 10 mM phosphate buffer pH 8.3 and then sonicated for 30 min. Stock solutions of extracts were prepared by dissolving 5 mg of each extract in the same solvent used for extraction. Working sample solution was prepared by diluting the stock solution with 50% ethanol (1.5625–50 µg/mL). Angiotensin converting enzyme (ACE) was prepared at 0.05 U/mL in phosphate buffer pH 8.3 containing 0.1% bovine serum albumin (BSA). Then, 50 µL of purified ACE enzyme and 20 µL extracts, reference compounds or captopril at different concentrations were added to 130 µL of HHL substrate. Control reaction mixture was contained 20 µL of 50% ethanol instead of extracts. All samples were incubated for 2 h at 37 °C. The reaction was stopped by heating the samples at 100 °C. All samples were analyzed by HPLC after adding 800 µL of water. ACE inhibition rate was calculated by the following equation:

ACE inhibition activity of test substances (%) = [(HA concentration levels without test substances) − (HA concentration levels with test substances)/(HA concentration levels without test substances)] × 100
(3)

All analyses were performed thrice and their results are presented as the mean ± standard deviation (SD). If the test substance displayed inhibition activity toward ACE, these values would be increased significantly (up to 100%). On the other hand, if the substance did not display inhibition activity, these values would only be altered slightly by the test substances.

### 4.6. Chelation of Zinc Ion (II) by OS-E, RA, TMF, SIN, EUP and Captopril

Capacity of zinc Ion (II) chelation by captopril, RA, TMF, SIN, EUP and OS-E was determined using a method described by Karamać [50]. Briefly, 0.01 M hexamine/HCl buffer was prepared at pH 5.0 with addition of 0.01 M KCl. Stock solution of captopril. RA, TMF, SIN, EUP, and OS-E were prepared in hexamine/HCl at concentration of 5 mg/mL. A series of working sample solutions were prepared by diluting the stock solutions with hexamine/HCl buffer (0–5 mg/mL). ZnCl_2_ was prepared in the same buffer at concentration of 0.8 mM. Then, 1 mL of sample solutions was mixed with 1 mL ZnCl_2_ solution and 0.1 mL tetramethylmurexide at concentration of 1 mM. Absorbance was recorded at 462 nm and 530 nm and then the ratio of A462/A530 was calculated. Distilled deionized water was used instead of tetramethylmurexide reagent in control samples. A standard curve of absorbance ratio versus metal ions concentrations (0 to 3.2 mM) was prepared and the percentage of bound metal ions was calculated.

Percentage of bond with Zn^2+^ = 1 − (Concentration of free Zn^2+^/Concentration of total Zn^2+^) × 100 (4)

### 4.7. Molecular Docking Study

3D Ligand structure of RA, TMF, SIN, EUP, and captopril (positive control) were drawn in ChemBio Draw and 3D Protein structure of Angiotensin Converting Enzyme (ACE; PDB:1O86) were downloaded from RCSB protein data bank. RA, TMF, SIN, EUP and captopril structures were further studied for their possible binding with active site of ACE enzyme, which is Zn^2+^ or protein–ligand, by docking studies using *LeadIT-FlexX*: BioSolveIT’s LeadIT program. 

*LeadIT-FlexX*: BioSolveIT’s LeadIT program was used to dock the compounds as flexible function of FlexX algorithm [51]. This program detects the binding site according to reference ligand by superimposition of the experimented ligand. In addition, active site was defined by selecting the residue of the protein with 10 Å on the center of the ligand. Top 10 poses were selected to analyze the free binding energy (ΔG) of the protein–ligand complex and ligand efficiency using Hyde assessment [52].

## 5. Conclusions

In this study, we have successfully developed and validated a new HPLC-UV method for measurement of ACE activity in OS plant extracts and its reference compounds (RA, TMF, SIN and EUP). Moreover, we have demonstrated that changes in the flavonoid active core affect its capacity to inhibit the ACE, and production of chelate with Zn (II) ion. In addition, docking study on the reference compounds of OS also confirmed that the RA, TMF, SIN and EUP present in OS extracts are able to inhibit ACE via interaction with Zn (II) ion and this interaction is stabilized by other interactions with amino acids in the active site. Therefore, the information provided in this study can be useful for designing new ACE inhibitors based on flavonoids compounds.

## Figures and Tables

**Table 1:** Quantification of four marker compounds in OS extracts. Results are shown as average (mg/g; marker compound/extract).

Component	Water Extract (OS-W)	Ethanolic Extract (OS-E)	Methanolic Extract (OS-M)	50% Ethanolic Extract (OS-EW)	50% Methanolic Extract (OS-MW)
RA	31.0 ± 0.2	219.8 ± 0.6	200.8 ± 1.5	235.5 ± 0.2	212.3 ± 0.01
TMF	0.0 ± 0.7	11.5 ± 1.0	0.0 ± 0.2	4.3 ± 0.01	0.9 ± 0.01
SIN	3.2 ± 0.01	23.2 ± 0.1	6.1 ± 0.01	10.5 ± 0.01	8.0 ± 0.3
EUP	2.3 ± 0.01	166.9 ± 3.5	22.4 ± 0.3	66.8 ± 0.9	30.6 ± 0.1

**Table 2:** The half-maximal inhibitory concentration (IC_50_) of OS extracts, standard compounds and captopril on in vitro ACE inhibitory assay, each value represents mean ± SD (*n* = 3).

Components	IC_50_	Unit
OS-W	358.8 ± 24.2	µg/mL
OS-E	45.8 ± 1.2	µg/mL
OS-M	63.7 ± 1.1	µg/mL
OS-EW	58.1 ± 2.0	µg/mL
OS-MW	78.2 ± 7.9	µg/mL
Captopril	0.002 ± 0.001	µM
RA	18.8 ± 0.2	µg/mL
TMF	27.9 ± 2.4	µg/mL
SIN	29.5 ± 0.3	µg/mL
EUP	15.4 ± 4.5	µg/mL

**Table 3:** Summary of the docking scores and reported binding affinities of RA, SIN, TMF and EUP compounds with ACE in the docking analysis with LeadIT FlexX Scoring functions.

Receptor-Ligand Complex (LEADIT)	Binding Affinity Energy, ΔG (kJ/mol)	Ligand Efficiency (kcal/mol Per Heavy Atom)	Residue Interactions	Hydrogen Bonds
ACE-RA	−17	0.16	Zn701, Tyr523, Glu384, His353, Ser355, Val516, Phe512, Asn70, His353, Hoh2570, His383 and Val380	6
ACE-SIN	10	0	Zn701, Ser355, Phe512, His383, Gln281, Lys511, Tyr523, His353, His513 and Val518	4
ACE-TMF	−6	0.06	Zn701, His383, Tyr523, Hoh2409, Gln281, Gly2000, Lys511, His353, Ser355, Phe512, His513 and Hoh2570.	5
ACE-EUP	−29	0.28	Zn701, Hoh2570, Ser355, His353, Val518, Phe512, His513, Gln281, Lys511, His513 and Tyr523	7
ACE-Captopril	−38	0.64	Zn701, Tyr523, Tyr520, His513, Lys511, Gln281, His353 and His383	7
